# Enhancing Hot-Electron Photodetection of a TiO_2_/Au Schottky Junction by Employing a Hybrid Plasmonic Nanostructure

**DOI:** 10.3390/ma15082737

**Published:** 2022-04-08

**Authors:** Wenyan Wang, Cheng Zhang, Kaifang Qiu, Guohui Li, Aiping Zhai, Yuying Hao, Xiaofeng Li, Yanxia Cui

**Affiliations:** 1 College of Physics and Optoelectronics, Key Laboratory of Advanced Transducers and Intelligent Control System of Ministry of Education, Key Laboratory of Interface Science and Engineering in Advanced Materials, Taiyuan University of Technology, Taiyuan 030024, China; wangwenyan@tyut.edu.cn (W.W.); qiukf_1995@163.com (K.Q.); liguohui@tyut.edu.cn (G.L.); haoyuying@tyut.edu.cn (Y.H.); 2 School of Optoelectronic Science, Engineering & Collaborative Innovation Center of Suzhou Nano Science and Technology, Key Laboratory of Advanced Optical Manufacturing Technologies of Jiangsu Province & Key Laboratory of Modern Optical Technologies of Education Ministry of China, Soochow University, Suzhou 215006, China; zhangc@suda.edu.cn (C.Z.); xfli@suda.edu.cn (X.L.)

**Keywords:** plasmonic, hot-electron, photocurrent, nanostructure, photodetector

## Abstract

Hot-electron photodetectors (HEPDs) are triggering a strong surge of interest in applications of image sensors and optics communication, since they can realize photoelectric responses when the incident photon energy is lower than the bandwidth of the semiconductor. In traditional HEPD systems, the metal layers are dressed with regular gratings, which can only excite plasmonic resonance over a narrow bandwidth, limiting the hot-electron photoelectric effect. To break this limitation, hybrid plasmonic nanostructures should be applied in HEPDs. Here, we propose a TiO_2_ based HEPD device incorporated with a hybrid plasmonic nanostructure, which consists of Au nanoparticles (Au NPs) and a conformal transparent Au film. With the assistance of the plasmonic resonances excited in this hybrid nanostructure, the spectrum of the photocurrent response is significantly broadened from the UV band to the visible and near-infrared (NIR) ranges. It is demonstrated that at the wavelengths of 660 nm and 850 nm, the photocurrent in the hybrid HEPD device is enhanced by 610% and 960%, respectively, compared with the counterparts without the addition of Au NPs. This work promotes the development of high performances HEPDs, offering an alternative strategy for realizing photodetection and image sensing in the NIR range.

## 1. Introduction

Traditional photodetectors use semiconductors to sense light, covering limited wavelength ranges because the semiconductor films can only absorb light with a photon energy above their bandgaps [1]. In order to break the limitation, metals which can absorb light over a broadband wavelength range have been adopted as the medium to sense light. Metals with different structure or material properties also have also been widely applied in other optoelectronic devices, e.g., solar cells and memory devices [2,3,4,5,6,7,8]. In metals such as gold (Au), silver (Ag) and so on, the absorbed photons can be transformed into hot carriers, i.e., hot electrons and hot holes, of which the energy is higher than the Fermi level [9,10,11,12,13]. Before the carriers are relaxed, an appropriate metal/semiconductor Schottky junction enables the transfer of hot carriers from the metal to the semiconductor [14,15,16,17]. The carriers entering the semiconductor can then be collected by the external electrode, which results in a photocurrent. Because this kind of device collects the hot carriers excited by photons in metals, they are called hot carrier photodetectors. A metal/n-type semiconductor Schottky junction collects the hot electrons and a metal/p-type semiconductor one collects the hot holes, forming the hot electron photodetectors (HEPDs) [18] and hot hole photodetectors [19,20], respectively. Up to now, HEPDs based on n-type silicon, TiO_2_, ZnO, etc., have been frequently researched [16,21,22,23].

The early HEPDs are based on planar metal films, which have limited absorption efficiency, resulting in inefficient photoelectric conversion [19]. It is known that the absorption efficiencies of light in metal can be significantly enhanced through the decoration of gratings, which can excite plasmonic resonances. Recently, a series of regular plasmonic grating-based HEPDs have been proposed to achieve enhanced hot electron photoelectric responses [3,18,24,25]. For example, Knight et al. demonstrated a Si-based HEPD with an embedded one-dimensional plasmonic grating, increasing the responsivity at 1.5 μm by 25 times in contrast with the planar device [26]. Wu et al. proposed an Au—ZnO—Au type HEPD with one-dimensional conformal plasmonic grating, which shows a peak responsivity of 0.032 mA/W at 682 nm, which is three times greater than that with the conventional grating [27]. Feng et al. introduced two regular plasmonic gratings into a Si nanowire-based HEPD, yielding a peak responsivity of 94.5 mA/W at 1.15 μm under transverse magnetic illumination [28]; in contrast, the control device without plasmonic gratings showed no photocurrent response at that wavelength.

Although significant advances have been made, most of reported HEPD devices based on regular metal gratings still bear unsatisfactory photoelectric effects because the plasmonic resonances can only be excited over narrow bandwidths. To increase the absorption of light over broadband wavelength range in HEPD devices, irregular plasmonic nanostructures could be adopted. It should be mentioned that irregular Au nanostructures formed on TiO_2_ films by annealing have been utilized to improve hydrogen production by photodissociation of water in photocatalytic applications [29,30]. Inspired by those works, we propose a TiO_2_-based HEPD device with an architecture of FTO/TiO_2_/Au NP/Au film (FTO: Fluorine-Doped Tin Oxide). The device employs a hybrid plasma nanostructure as the generation and emission source of hot electrons, which consists of Au nanoparticles (Au NPs) and a conformal transparent Au film, prepared by depositing thin Au films onto uneven TiO_2_ layer-loaded Au NPs. Here, the TiO_2_ acts as a receiving and transmitting material for hot electrons. The uneven TiO_2_ layer-loaded Au NPs are fabricated by depositing thin Au films onto a TiO_2_ layer, followed by thermal annealing. Compared with the reference HEPD device with the transparent Au film being directly coated on the TiO_2_ layer (FTO/TiO_2_/Au film), the HEPD device with the hybrid plasmonic nanostructure possesses improved absorption of light over a broadband wavelength range from UV to near-infrared (NIR). Also, the absorption performance of the proposed HEPD device is much superior to that of the multilayer film comprised of FTO/TiO_2_/Au NPs. From the transient photocurrent characterizations, it is found that photocurrent in the hybrid plasmonic HEPD device is significantly enhanced compared with that in the reference device over the wavelength range from UV to NIR. Specifically, at 660 nm and 850 nm, the photocurrent of the hybrid plasmonic HEPD device is enhanced by 610% and 960%, respectively, compared with that of the reference device without Au NPs. Simulation further verifies that the enhanced photocurrent response over the broadband wavelength range is ascribed to the excitation of diverse plasmonic resonances in this Au NP/Au film hybrid plasmonic nanostructure. This approach can also be applied to improve the performance of HEPDs made of other semiconductors. Our work contributes to the development of HEPDs, which might offer an alternative photodetection strategy in certain extreme conditions where the photoelectric effects in traditional semiconductors lose their effectiveness.

## 2. Structure and Experimental Details

**Figure 1:**
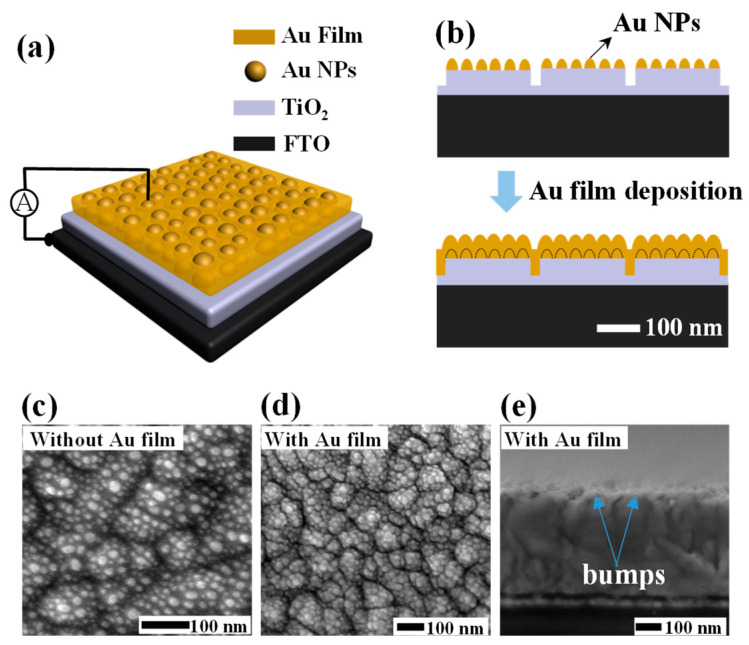
(**a**) 3D schematic diagram of the proposed plasmonic HEPD with a configuration of FTO/TiO_2_/Au NP/Au film. (**b**) 2D cross-sectional diagram of the HEPD illustrating the conformal deposition process for preparing the transparent Au film. (**c**,**d**) Top-view SEM images of the FTO/TiO_2_/Au NP multilayer sample and the proposed HEPD. (**e**) Cross-sectional SEM image of the proposed HEPD.

The proposed hybrid plasmonic nanostructure based HEPD has an architecture as shown in Figure 1a, and the related preparation scheme in experiment can be found in Figure S1 of Supplementary Materials (SUPP). During the fabrication, the wet-cleaned FTO glass substrates were first subjected to the surface plasma treatment to increase the work function of FTO substrates. Then, TiO_2_ and Au films were prepared by radio frequency (RF) and direct current (DC) magnetron sputtering, respectively. In detail, a TiO_2_ layer with a thickness of 20 nm was deposited onto the FTO substrate, followed by the deposition of an ultrathin Au film with its nominal thickness (*t_n_*) varying from 2 to 8 nm. Then, the as-prepared multiplayer samples were annealed in air at 500 °C. The annealing process could, simultaneously, transform the ultrathin Au film into a layer of Au NPs, and transform the amorphous TiO_2_ film into its polycrystalline anatase film structure with a rough profile. After that, another thin Au film was deposited onto the annealed samples by DC magnetron sputtering. Here, the thin Au film can act as the transparent electrode, with its thickness fixed to 20 nm. The photodetector device without the Au NPs was also fabricated as the reference HEPD by directly sputtering a 20 nm thick Au film on the annealed TiO_2_ film.

From the surface morphology characterization of the FTO/TiO_2_/Au NP multilayer sample by scanning electron microscopy (SEM, Hitachi SU8010, Hitachi, Tokyo, Japan) as shown in Figure 1c, it was witnessed that the Au film transformed into a layer of Au NPs after the annealing process, similar to the observations in Refs. [31,32]. The presence of large fluctuations in the background indicated that the bottom anatase TiO_2_ film was uneven, and bumps with sizes of hundred nanometers appeared on its surface. This means that on top of one big TiO_2_ bump, there were multiple small Au NPs. Figure 1d shows the SEM characterization of the FTO/TiO_2_/Au NP/Au film sample, which reflects that this complicated profile can be maintained after the deposition of the top of the Au electrode film. From the cross-sectional SEM image of the FTO/TiO_2_/Au NP/Au film sample as shown in Figure 1e, the TiO_2_ bumps can also be clearly observed. The surface topographies of both the FTO/TiO_2_/Au NPs and FTO/TiO_2_/Au NPs/Au film samples have also been characterized by atomic force microscope (AFM) (Park NX10, Park, Korea) (shown in Figure S2 of SUPP), which indicate that the topographies bear negligible differences, proving that the conformal film deposition process as illustrated by the diagram in Figure 1b has been carried out.

## 3. Results and Discussions

**Figure 2:**
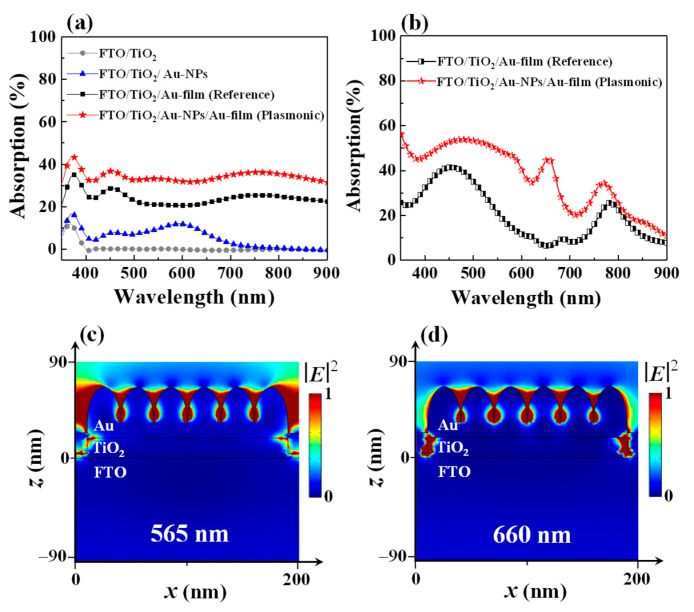
(**a**) Absorption spectra of the hybrid plasmonic and reference HEPDs with the configurations of FTO/TiO_2_/Au NP/Au film and FTO/TiO_2_/Au film, respectively. For comparison, the absorption spectra of the multilayer structures with the configurations of FTO/TiO_2_ and FTO/TiO_2_/Au NPs are also displayed. (**b**) Simulated absorption spectra of the hybrid plasmonic and reference HEPDs. Simulated maps of electric filed amplitude |*E*|^2^ of the hybrid plasmonic at the wavelengths of 565 nm (**c**) and 660 nm (**d**).

Figure 2a shows the absorption spectra of the hybrid plasmonic and reference HEPDs, measured using a UV-VIS-NIR spectrometer (Agilent Cary 5000, Agilent, China). For comparison, the absorption spectra of the two multilayer structures with the configurations of FTO/TiO_2_ and FTO/TiO_2_/Au NPs were also measured, as shown in Figure 2a. As one can see from the curve with dots in Figure 2a, the bare TiO_2_ film can only absorb light with a wavelength shorter than 400 nm, which is determined by its energy band gap. After introducing a layer of Au NPs on top of the TiO_2_ layer, the absorption band can be broadened to the visible range, with an average absorption efficiency of 5.66% (calculated over the range from 350 nm to 900 nm); see the curve with triangles in Figure 2a. There are two absorption peaks produced at 460 nm and 595 nm, induced by the plasmonic resonances of Au NPs. Furthermore, significant improvement of the absorption performances—including both the absorption spectrum range and the absorption efficiency—can be acquired when an additional thin Au electrode layer is deposited on top of the layer of Au NPs, forming a hybrid plasmonic HEPD with *t_n_* = 4 nm. It can be seen from the curve with stars in Figure 2a that the hybrid plasmonic HEPD can absorb not only UV and visible light, but also NIR light. Over the visible and NIR wavelength range, the hybrid plasmonic HEPD exhibits multiple absorption peaks, arising from the collective plasmonic resonances of hybrid Au NP/Au film nanostructures. As a result, the average absorption efficiency from 350 to 900 nm reaches 34.3%, which is enhanced by 506% with respect to the FTO/TiO_2_/Au NPs sample. One can also see from the curve with squares in Figure 2a that the reference HEPD (the curve with stars) also displays a broad absorption spectrum over the wavelength range from 350 nm to 900 nm, with an average absorption efficiency of 24.2%. In contrast with the reference HEPD, the hybrid plasmonic HEPD possesses a 41.7% higher average absorption efficiency; therefore, it is anticipated that the hybrid plasmonic HEPD would advance the reference in the aspect of hot carrier generation, thereby giving rise to an enhanced photocurrent.

In order to clarify the broadband absorption performance of the hybrid plasmonic HEPD along with its superiority on the absorption efficiency over the reference, the optical simulations are carried out by the three-dimensional (3D) Finite Element Method (FEM) [7]. Here, both the absorption spectra and maps of electric filed are monitored for analysis. It is known that a flat Au film would not produce other surface plasmon resonances under normal illumination, except for the bulk plasma. The absorption peaks at the wavelengths of ~450 and ~800 nm of the reference device indicate that the top surface of the FTO/TiO_2_ film for the following Au film deposition is not flat. This is in agreement with the uneven TiO_2_ morphologies observed from Figure 1c–e. Based on this fact, in simulation, we simplify the hybrid plasmonic and reference HEPDs into the structures with uneven TiO_2_ surfaces, as shown by Figure 1b. Here, the lattice constant is defined to 200 nm, and the width and height of TiO_2_ bump are set to 180 nm and 15 nm, respectively. The dimensions of the Au NPs array are specified with a height of 30 nm and radius of 15 nm. In simulation, the wavelength-dependent refractive indices and extinction coefficients of all materials are used [33,34]. The calculations are carried out assuming a periodic boundary along the *x*- and *y*-axes. Perfectly matched layer (PML) boundaries are applied to the top and bottom surfaces. Light is illuminated from the Au transparent electrode side.

Figure 2b shows the representative calculated absorption spectra of the hybrid plasmonic HEPD and the reference device. It is witnessed that the hybrid plasmonic HEPD exhibits improved absorption over the entire wavelength range of interest, in contrast with the reference HEPD. At the wavelengths of ~450 and ~800 nm, both the hybrid plasmonic HEPD and the reference devices display strong absorption peaks, which could be attributed to the bulk plasma excitation of Au material and the plasmonic resonance excited by the Au grooves located between neighboring TiO_2_ bumps, respectively. The introduction of Au NPs is responsible for increasing the absorption efficiencies of these two wavelengths bands (~450 and ~800 nm). Incorporating Au NPs can produce additional plasmonic resonances, e.g., at 565 nm and 660 nm, with the electric field maps (|*E*|^2^) as shown in Figure 2c,d. At 565 nm, the strong absorption is mainly induced by the hybridization of the plasmonic resonances excited by the arrayed Au NPs and the Au grooves located between neighboring TiO_2_ bumps. At 660 nm, the plasmonic resonances excited by the arrayed Au NPs play a major role in capturing light, while the resonance of the Au grooves located between neighboring TiO_2_ bumps is quite weak. It should be mentioned that for the as-prepared samples, the Au NPs vary from each other in size, shape, and distance, and the TiO_2_ bumps are also distinct from each other, as shown in Figure 1c, which caused the measured absorption efficiency of the hybrid plasmonic HEPD to display a weak dependence on wavelength. In short, the hybrid plasmonic HEPD enriches the excitation of plasmonic resonances over a broadband wavelength range, which can more efficiently capture the incident light in contrast with the reference HEPD, favoring the subsequent hot carrier generation.

**Figure 3:**
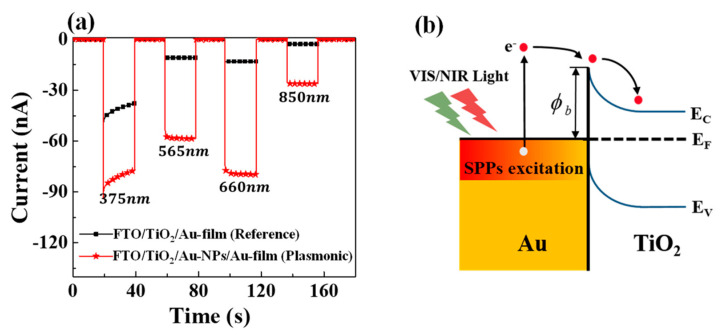
(**a**) Measured transient photocurrent responses (*I-t* curves) of the hybrid plasmonic and reference HEPDs when the wavelength of the incident LED source is varied (*λ* = 375 nm, 565 nm, 660 nm, and 850 nm). (**b**) Schematic diagram of the hot electron transfer processes over the Schottky barrier under illumination by visible/NIR light.

Next, we compare the photocurrent performances of the hybrid plasmonic and reference HEPDs when the wavelength of the incident light is tuned (*λ* = 375 nm, 565 nm, 660 nm, and 850 nm, as displayed in Figure 3a). The transient current (*I*-*t*) characteristics of the two HEPDs were measured under illumination by LED lamps with the power density fixed to 10.2 mW/cm^2^, using the semiconductor analyzer (Agilent B1500A, Keysight Technologies, Malaysia). The photosensitive area of HEPDs is defined by the intersection of the top and down electrodes (0.04 cm^2^). It should be noted that all photo responses are measured under zero bias, since applying a bias would induce the hot carrier signal to be submerged in the injected background carriers. The direction of current flow from the Au electrode towards the FTO electrode is specified as positive. Thus, the acquired negative currents under illumination in Figure 3a confirm that the hot electrons successfully transfer into the TiO_2_ layer from the adjacent Au. The schematic diagram of this hot electron transfer process is illustrated in Figure 3b. As shown in Figure 3a, at the four investigated wavelengths, the photocurrents of the hybrid plasmonic HEPD device at four wavelengths are much higher than their individual references. Here, we obtain a photocurrent enhancement factor of 610% at 660 nm and 960% at 850 nm, and the light to dark current ratio of the hybrid plasmonic HEPD at 660 nm reaches 1747. For the hybrid plasmonic HEPD, the stronger absorption in the adjacent Au layer (Au NPs plus the Au film) can account for the significantly improved photocurrent. It can be noticed that the photocurrent enhancement factor is much greater than the absorption enhancement factor (41.7%) derived from Figure 2a. This could be ascribed to the non-uniform hot carrier transfer rates in different regions, which can induce variation in the subsequent carrier collection efficiency. It is deduced that the opportunities of hot carriers generated on the arrayed Au NPs transferring into the TiO_2_ layer are greater than those of hot carriers generated in the Au grooves between neighboring TiO_2_ bumps. It can also be observed that the photocurrent enhancement factors at the visible and NIR ranges are much stronger than that at the UV wavelength (375 nm). The relatively weak photocurrent enhancement factor at 375 nm is because the photocurrent generated by TiO_2_ intrinsic absorption contributes to a current that flows with the direction opposite to that of the hot carrier current.

**Figure 4:**
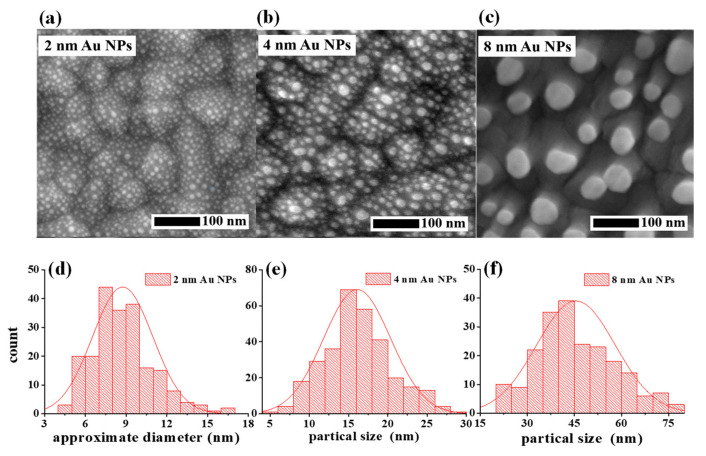
(**a**–**c**) Top-view SEM images of the FTO/TiO_2_/Au NP multilayer samples when the nominal thickness *t_n_* of the Au film subjected to the thermal treatment is tuned. (**d**–**f**) Histograms of the average size of the Au NPs with tuned *t_n_*.

**Figure 5:**
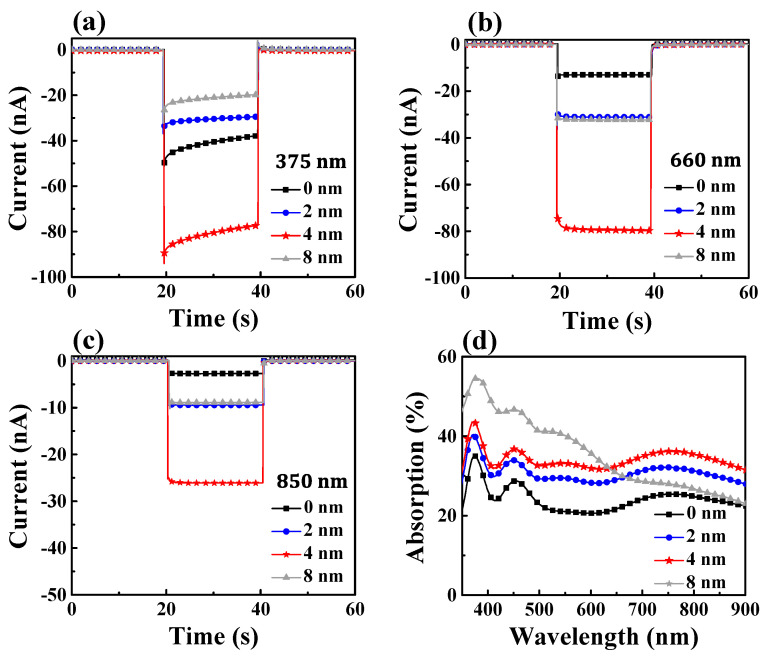
Transient photocurrents of the hybrid plasmonic HEPDs depending on varied Au NPs (by tuning *t_n_* =0, 2, 4, and 8 nm) under illumination of 375 nm (**a**), 660 nm (**b**), and 850 nm (**c**) light. (**d**) Measured absorption spectra of the HEPDs with different Au NPs.

Lastly, we studied the photocurrent responses of the hybrid plasmonic HEPD influenced by Au NPs. The geometries of Au NPs are tuned by adjusting the nominal thickness (*t_n_*) of the Au film with the fixed thermal annealing treatment process. Figure 4a–c shows the top-view SEM images of different Au NPs deposited on the TiO_2_ layer, obtained by annealing Au films with *t_n_* = 2 nm, 4 nm, and 8 nm, respectively. The related histograms of the nanoparticle size distribution are plotted in Figure 4d–f, respectively. It can be seen that the Au film thickness plays a dominant role in affecting the sizes and densities of the annealed Au NPs. With the increase of the Au film thickness, Au NPs with greater sizes and smaller densities are inclined to be formed. Here, it is found that the mean diameters of Au NPs when *t_n_* = 2 nm, 4 nm, and 8 nm are about 9 nm, 15 nm, and 45 nm, respectively. The acquired Au NPs with various morphologies cause the hybrid plasmonic HEPD devices to perform differently in absorption, as shown in Figure 5d. One can clearly sees that both the densely-distributed Au NPs with *t_n_* = 2 nm and *t_n_* = 4 nm can bring forward significant absorption enhancement over a broadband spectrum range. The condition of *t_n_* = 4 nm corresponds to the optimized effect on enhancing absorption, indicating that the Au NPs with an average size of 15 nm can induce stronger plasmonic resonances over a broadband wavelength range than those 9 nm-sized Au NPs. In contrast, the over large, sparsely-distributed Au NPs obtained by 8 nm thick Au film can only enhance the absorption of light at a wavelength range shorter than 600 nm. It is deduced that the Au NPs as shown in Figure 4c are too sparsely distributed, which means the plasmonic resonances arising from the intense coupling effects between neighboring Au NPs as displayed in Figure 2c,d can no longer be excited. This could explain the observed narrow absorption enhancement spectrum for *t_n_* = 8 nm in Figure 5d. It should be mentioned that the crystallinity of Au also contributes to the work function of Au film. Thus, the height of the Schottky barrier will be slightly tuned if the crystallinity of Au is changed, giving rise to the variation of the photodetector performances. In this work, because all samples are annealed at the same temperature, the impact of Au crystallinities on samples is neglected.

Figure 5a–c shows the wavelength-dependent transient photocurrent responses when the Au NPs in the hybrid plasmonic HEPD are tuned through adjusting *t_n_*; the corresponding photocurrents of the reference HEPD (i.e., *t_n_* = 0 nm) are also added for comparison. One can see that at varied wavelengths of 375 nm, 660 nm, and 850 nm, the hybrid plasmonic HEPD device with *t_n_* = 4 nm outputs the highest photocurrent, in agreement with the results of absorption displayed in Figure 5d. Compared with the optimized HEPD device, the device with *t_n_* = 2 nm is inferior in photocurrent performance, likely owing to its relatively poor absorption. Although the device with *t_n_* = 8 nm has a stronger absorption enhancement factor at the short wavelength range, its photocurrent is even lower than that of the reference at 375 nm; this could be due to the bulk plasma excitation in the Au material. For the device with 45 nm-sized Au NPs, a large portion of the absorbed energy at the wavelength of 375 nm would dissipate into non-radiative losses due to the plasma excitation, instead of generating plasmonic hot carriers.

## 4. Conclusions

In summary, we have demonstrated a TiO_2_-based HEPD by incorporating a hybrid plasmonic nanostructure made of Au NPs together with a conformal Au film. Different from other similar approaches that were designed for high-efficiency hydrogen generation in the photocatalysts, this hybrid plasma nanostructure was used in the broadband sensitive photodetector. It can fully realize the diversity of structural parameters and excite rich surface plasmon resonance, enabling the device to respond sensitively to the incident photons over a broadband wavelength range, covering UV, visible, and NIR. It is shown that the photocurrent of the hybrid plasmonic HEPD is much superior to that of the reference HEPD without employing Au NPs. At the wavelengths of 660 nm and 850 nm, the photocurrent in the hybrid plasmonic HEPD device is enhanced by 610% and 960%, respectively, compared with that in the reference. Our systematical characterizations combined with the simulation analysis have explained that the photocurrent enhancement is a product of the excitation of diverse plasmonic resonances in this hybrid plasmonic nanostructure. The nominal thickness of the Au film subjected to thermal annealing plays a dominant role in affecting the morphologies of the incorporated Au NPs, which further determines the photocurrent signals of the plasmonic HEPD devices. It is concluded that utilizing the Au film with a nominal thickness of 4 nm, allowing the formation of closely packaged Au NPs with an average size of 15 nm, produced the most efficient light absorption and photocurrent generation over the broadband wavelength range from UV to NIR. This work paves the way for the development of hot carrier photodetectors, which might open up applications in some extreme conditions where the photoelectric effects induced by semiconductor absorption lose their effectiveness.

## Data Availability

Data underlying the results presented in this paper are not publicly available at this time but may be obtained from the authors upon reasonable request.

## Supplementary Materials

The following supporting information can be downloaded at: https://www.mdpi.com/article/10.3390/ma15082737/s1, Figure S1: Scheme of the proposed HEPD device preparation in experiment; Figure S2: (a) The top-view AFM images. (a) for the structure of FTO/TiO_2_/Au NPs, (b) for the proposed structure of FTO/TiO_2_/Au NPs/Au films.
