# Supplementary material for: Enhancing Hot-Electron Photodetection of a TiO2/Au Schottky Junction by Employing a Hybrid Plasmonic Nanostructure
Source: Materials (Basel). 2022 Apr 8;15(8):2737. doi: 10.3390/ma15082737 (PMC9025816; doi:10.3390/ma15082737)
Supplement: Supplementary file 1 [file materials-15-02737-s001.zip › materials-1639858-supplementary.pdf]

materials

Enhancing Hot-electron Photodetection of a TiO_2/Au Schottky Junction by Employing a Hybrid Plasmonic Nanostructure: Supplementary Material

Wenyan Wang [1], Cheng Zhang [2], Kaifang Qiu [1], Guohui Li [1], Aiping Zhai [1,*], Yuying Hao [1], Xiaofeng LI [2] and Yanxia Cui [1,*]

[1] College of Physics and Optoelectronics, Key Laboratory of Advanced Transducers and Intelligent Control System of Ministry of Education, Key Laboratory of Interface Science and Engineering in Advanced Materials, Taiyuan University of Technology, Taiyuan 030024, China; wangwenyan@tyut.edu.cn (W.W.); qiukf_1995@163.com (K.Q.); liguohui@tyut.edu.cn (G.L.); haoyuying@tyut.edu.cn (Y.H.)
[2] School of Optoelectronic Science, Engineering & Collaborative Innovation Center of Suzhou Nano Science and Technology, Key Laboratory of Advanced Optical Manufacturing Technologies of Jiangsu Province & Key Laboratory of Modern Optical Technologies of Education Ministry of China, Soochow University, Suzhou 215006, China; zhangc@suda.edu.cn (C.Z.); xfli@suda.edu.cn (X.L.)
* Correspondence: aipz@foxmail.com (A.Z.); yanxiacui@tyut.edu.cn (Y.C.)

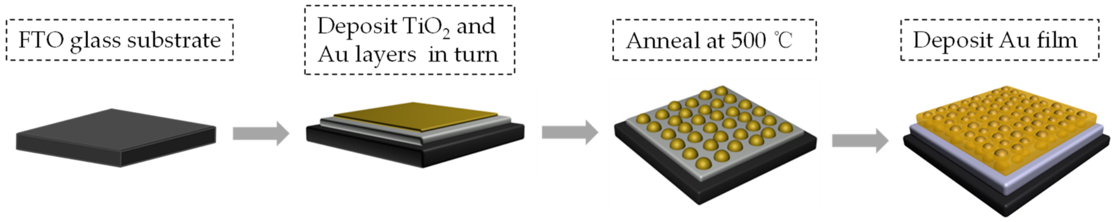

Figure S1. Scheme of the proposed HEPD device preparation in experiment.

Figure S1 shows the preparation scheme of the proposed HEPD device in experiment. During the fabrication, the wet-cleaned FTO glass substrates were first subjected to the surface plasma treatment for increasing the work function of FTO substrates. Then, TiO_2 and Au films were prepared by radio frequency (RF) and direct current (DC) magnetron sputtering, respectively. Then, the as-prepared multiplayer samples were annealed in air at 500 °C for 3 hours. The annealing process could, simultaneously, transform the ultrathin Au film into a layer of Au NPs, and transform the amorphous TiO_2 film into its polycrystalline anatase film structure with a rough profile. After that, another thin Au film was deposited onto the annealed samples by DC magnetron sputtering, to complete the preparation of the HEPD device proposed in this paper.

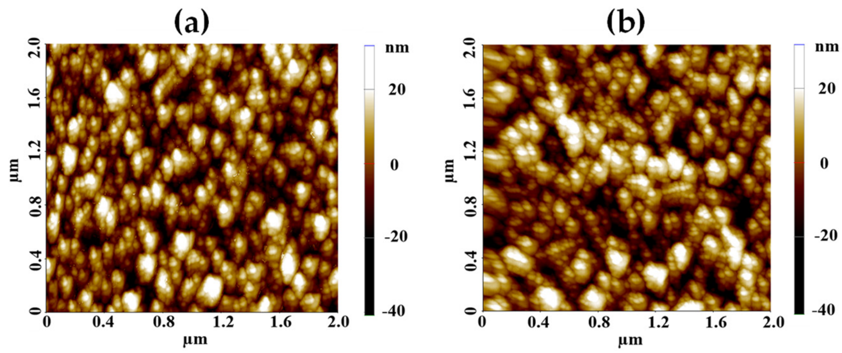

Figure S2. The top-view AFM images. (**a**) for the structure of FTO/TiO_2/Au NPs, (**b**) for the proposed structure of FTO/TiO_2/Au NPs/Au films.

Figure S2 shows the top-view AFM images of the sample FTO/TiO_2/Au NPs and the proposed HEPD respectively. It can be seen that their surface roughness is almost the same, showing obvious conformal characteristics.
